# Clinical Analysis of Macular Choroidal Thickness in Pseudoexfoliative Glaucoma and Primary Open-Angle Glaucoma

**DOI:** 10.1155/2021/3897952

**Published:** 2021-11-16

**Authors:** Fan Li, Yiming Huo, Lihua Ma, Qing Zhang, Hengli Zhang, Xiaowei Yan, Yulei Geng, Guangxian Tang

**Affiliations:** ^1^Department of Ophthalmology, Shijiazhuang People's Hospital, 365 Jianhua Road, Shijiazhuang, Hebei, China; ^2^Department of Ophthalmology, Shijiazhuang Aier Eye Hospital, 252 Zhonghua Road, Shijiazhuang, Hebei, China

## Abstract

**Purpose:**

To evaluate the differences in macular choroidal thickness and volume among patients with pseudoexfoliative glaucoma (PXG), patients with primary open-angle glaucoma (POAG), and controls.

**Methods:**

A total of 50 PXG patients (50 eyes) and 56 POAG patients (56 eyes) were selected as the PXG group and the POAG group, respectively, in this case-control study. A total of 54 age-, gender-, IOP-, and axial length-matched healthy individuals (54 eyes) were selected as the control group. Enhanced-depth imaging-optical coherence tomography (EDI-OCT) was used to measure and analyze the choroidal thicknesses and volumes in 9 macular regions of all subjects.

**Results:**

The choroidal thicknesses in the central subfield (CSM), temporal inner macula (TIM), inferior inner macula (IIM), and temporal outer macula (TOM) and the mean macular choroidal thickness were significantly thinner in the PXG group than in the control group (all *P* < 0.05). The choroidal volumes in the TIM, IIM, and TOM and the mean macular choroidal volume were significantly smaller in the PXG group than in the control group (all *P* < 0.05). The choroidal thicknesses in the CSM and IIM and the mean macular choroidal thickness were significantly thinner in the PXG group than in the POAG group (all *P* < 0.05). The choroidal volumes in the IIM and TOM and the mean macular choroidal volume were significantly smaller in the PXG group than in the POAG group (all *P* < 0.05). Multivariable linear regression analysis showed that the mean macular choroidal thickness was significantly thinner in association with older subjects and longer axial length eyes. There was no association between the macular choroidal thickness of various macular regions and visual field mean defect (MD) in groups PXG and POAG (all *P* > 0.05).

**Conclusions:**

The macular choroidal thicknesses and volumes (inferior and temporal) in PXG patients were thinner and smaller than those in POAG patients and healthy individuals. The role of choroidal thickness changes in the course of PXG remains unclear. A future prospective study is needed to better define these changes in PXG patients.

## 1. Introduction

Glaucoma is a serious eye disease that can lead to blindness. The number of glaucoma cases in Asia is expected to increase from 39 million in 2013 to 111.8 million in 2040 [1]. Pseudoexfoliative glaucoma (PXG) is a type of secondary open-angle glaucoma caused by pseudoexfoliative syndrome (PEX) that accounts for approximately 25% of all cases of open-angle glaucoma. It is well known that patients with PXG have higher intraocular pressure (IOP), greater diurnal variation in IOP, slower retrobulbar blood flow, more severe visual field damage, and more rapid progression than patients with primary open-angle glaucoma (POAG) [2–5]. High IOP is a major risk factor for open-angle glaucoma. Controlling IOP can effectively delay the progression of glaucoma. However, in some cases, loss of visual function is exacerbated even when IOP is under control, indicating that there might be other factors that affect disease progression [6]. Choroidal and systemic blood flow parameters may play a role in the development and progression of glaucoma [7]. The measurement of choroidal thickness can provide important information about the rate of the choroidal blood flow. Spectral domain-optical coherence tomography (SD-OCT) can evaluate the choroid in vivo and offers high resolution and a fast scanning speed. The enhanced-depth imaging (EDI) mode can optimize OCT parameters, can image the full thickness of the choroid, and has high repeatability and reproducibility [8]. Previous studies on macular choroidal thickness in PXG and POAG have obtained mixed conclusions. One study has identified that patients affected by advanced POAG damage have a thinner choroidal thickness compared with normal subjects [6]. However, another study suggested that POAG was not significantly associated with a marked thinning or thickening of the choroid based on EDI-OCT measurements [9]. Dursun et al. [10] reported that macular and peripapillary choroidal thicknesses were decreased in PXG. In the present study, the macular choroidal thickness and volume in the eyes of Chinese patients with PXG and POAG were measured using EDI-OCT to investigate the changes in macular choroidal thickness in PEX and POAG eyes and to analyze the role of the choroid in the progression of PXG.

## 2. Materials and Methods

### 2.1. Patients

A total of 106 patients treated in our hospital between October 2018 and October 2020 were recruited for this study. The 50 PXG patients (50 eyes) were included in the PXG group, and 56 POAG patients (56 eyes) were included in the POAG group. Another 54 sex-, age-, IOP-, and axial length-matched healthy volunteers (54 eyes) were included in the control group. Both groups of glaucoma patients were treated with antiglaucoma medications to reduce IOP. There were no significant differences in age, sex, axial length, or IOP between the three groups (Table 1).

PXG diagnostic criteria were that the characteristic features of ocular PEX could be observed under a slit-lamp microscope, such as the appearance of gray-white exfoliative material at the pupillary margin, iris surface, and anterior lens capsule, IOP >21 mmHg, and glaucomatous optic nerve damage and visual field defects [11]. Diagnostic criteria for the normal control group were a normal-looking optic disc (no disc edge narrowing or optic disc hemorrhage), cup disc ratio (C/D) ≤ 0.3, binocular difference ≤0.2, IOP ≤21 mmHg, and normal examination of the visual field and chamber angle.

The inclusion criteria were as follows: (1) It meets the diagnostic criteria of PXG and POAG. (2) The age of the subjects was ≥60 years. (3) The equivalent spherical degree was ≤±6.0 D, and the cylindrical degree was ≤±3.0 D.

The exclusion criteria were other types of glaucoma (such as closed-angle glaucoma and secondary glaucoma); other ophthalmic diseases, such as corneal opacity, lens opacity, or other ocular diseases affecting the examination; previous history of ocular surgery or ocular trauma; retinal or macular diseases of the fundus; and systemic diseases such as hypertension and diabetes. This study followed the Helsinki Declaration and was approved by the ethics committee of Shijiazhuang People's Hospital (No.2018008). All subjects and their guardians signed informed consent forms.

### 2.2. Routine Examinations

All subjects underwent comprehensive eye examinations, including vision tests, slit-lamp microscopy, IOP measurement (Goldmann applanation tonometer), axial length measurement, gonioscopy, and fundus and visual field examinations.

### 2.3. OCT Procedure

**Figure 1:**
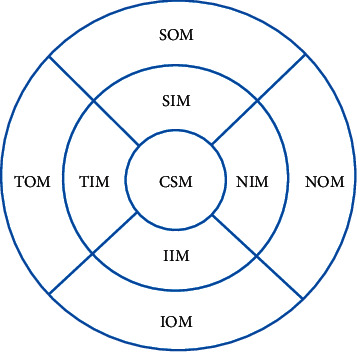
Measurement illustration of macular choroidal thickness at nine locations (reproduced from Li et al. [12]). CSM: central subfield macula; NIM: nasal inner macula; SIM: superior inner macula; IIM: inferior inner macula; TIM: temporal inner macula; NOM: nasal outer macula; SOM: superior outer macula; IOM: inferior outer macula; TOM: temporal outer macula.

**Figure 2:**
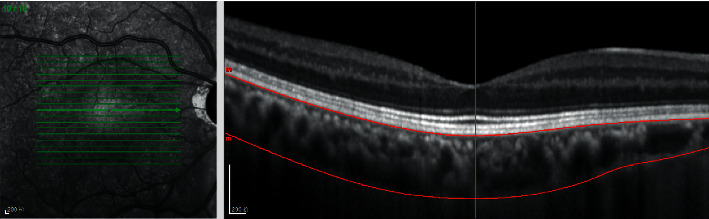
Optical coherence tomographic image (enhanced-depth imaging mode) for the measurement of the macular choroidal thickness.

All subjects underwent the SD-OCT (Spectralis HRA + OCT, Heidelberg, Germany). The measurement illustration of macular choroidal thickness is shown in Figure 1. The macular thickness and volume were scanned using the EDI mode of the SD-OCT macular thickness map examination procedure. For specific measurement methods, refer to previous studies [12]. On each scanned image, the inscribed segmentation line was labeled on the retinal pigment epithelium/Bruch membrane interface, and the outer segmentation line was placed on the scleral/choroidal interface to represent the internal and external choroidal boundaries, shown in Figure 2. The choroidal thickness measurements were performed by the same technician.

### 2.4. Visual Field Procedure

The visual fields of all subjects were examined using the SITA-Fast 30-2 examination procedure and a Humphrey-750i visual field analyzer (Carl Zeiss, Germany). The reliability criteria included a fixation loss rate of <20%, a false negative rate of <15%, and a false positive rate of <15%. Individuals who did not meet the criteria were excluded.

### 2.5. Statistical Analyses

Data were performed using SPSS 21.0 statistical software. The mean and standard deviations (M ± SD) of the above parameters were calculated. One-way ANOVA was performed for comparisons of age, axial length, IOP, visual field mean defect (MD), choroidal thickness, and volume among the three groups. An LSD *t*-test was used for pairwise comparisons. Multivariance linear regression analysis was done with stepwise modeling for determining the factors which influence mean macular choroidal thicknesses. Pearson's correlation analysis was used to analyze the correlation between macular thickness and visual field MD in PXG and POAG. Differences with *P* < 0.05 were considered statistically significant.

## 3. Results

The mean macular choroidal thicknesses of the PXG, POAG, and control groups were 171.81 ± 50.46 *μ*m, 193.56 ± 68.26 *μ*m, and 197.74 ± 46.82 *μ*m, respectively, and the mean macular choroidal volumes were 0.52 ± 0.15 *μ*m^3^, 0.59 ± 0.21 *μ*m^3^, and 0.60 ± 0.14 *μ*m^3^, respectively. The choroidal thicknesses in the central subfield (CSM), temporal inner macula (TIM), inferior inner macula (IIM), and temporal outer macula (TOM) and the mean macular choroidal thicknesses were significantly different among the three groups (*F* = 3.453, 4.195, 3.508, 5.407, and 3.149, respectively, all *P* < 0.05). The choroidal thicknesses in the nasal inner macula (NIM), superior inner macula (SIM), nasal outer macula (NOM), superior outer macula (SOM), and inferior outer macula (IOM) were not significantly different among the three groups (*F* = 2.371, 2.132, 2.468, 1.705, and 2.828, respectively, all *P* > 0.05). The choroidal volumes in the TIM, IIM, and TOM and the mean macular choroidal volumes were significantly different among the three groups (*F* = 4.208, 3.804, 6.393, and 3.264, respectively, all *P* < 0.05). The choroidal volumes in the CSM, NIM, SIM, NOM, SOM, and IOM were not significantly different (*F* = 2.340, 2.459, 2.141, 2.508, 1.765, and 2.650, respectively, all *P* > 0.05).

Pairwise comparisons showed that the choroidal thicknesses in the CSM, TIM, IIM, and TOM and the mean macular choroidal thickness were significantly thinner in the PXG group than in the control group (all *P* < 0.05) and that the choroidal volumes in the TIM, IIM, and TOM and the mean macular choroidal volumes were significantly smaller in the PXG group than in the control group (all *P* < 0.05). The choroidal thicknesses in the CSM and IIM and the mean macular choroidal thickness were significantly thinner in the PXG group than in the POAG group (all *P* < 0.05), and the choroidal thicknesses in the TIM and TOM were not significantly different between the two groups (all *P* > 0.05). The choroidal volumes in the IIM and TOM and the mean macular choroidal volume were significantly smaller in the PXG group than in the POAG group (all *P* < 0.05), and the choroidal volumes in the TIM were not significantly different between the two groups (*P* > 0.05). The choroidal thicknesses in the CSM, TIM, IIM, and TOM and the mean macular choroidal thickness were not significantly different between the POAG group and the control group (all *P* > 0.05), and the choroidal volumes in the TIM, IIM, and TOM and the mean macular choroidal volume were not significantly different between these two groups (all *P* > 0.05) (Table 2).

Multivariance linear regression analysis was done with stepwise modeling in order to investigate the factors which influence mean macular choroidal thickness in PXG and POAG groups. The influence of age and AL on mean macular choroidal thickness was found to be statistically significant. The mean macular choroidal thickness was significantly thinner in association with older subjects and longer AL eyes (*P* < 0.01, standardized regression coefficient = −0.377; *P*=0.01, standardized regression coefficient = −0.232).

There was no association between CSM, NIM, SIM, TIM, IIM, NOM, SOM, TOM, IOM, mean choroidal thickness, and visual field defects in group PXG (*r* = −0.081, *P*=0.575; *r* = −0.043, *P*=0.765; *r* = −0.141, *P*=0.330; *r* = −0.133, *P*=0.355; *r* = −0.032, *P*=0.826; *r* = 0.064, *P*=0.660; *r* = −0.069, *P*=0.635; *r* = −0.170, *P*=0.239; *r* = −0.072, *P*=0.618; *r* = −0.082, *P*=0.571). There was no association between CSM, NIM, SIM, TIM, IIM, NOM, SOM, TOM, IOM choroidal thickness, and visual field defects in group POAG (*r* = 0.244, *P*=0.071; *r* = 0.239, *P*=0.076; *r* = 0.271, *P*=0.054; *r* = 0.237, *P*=0.079; *r* = 0.158, *P*=0.243; *r* = 0.181, *P*=0.182; *r* = 0.242, *P*=0.072; *r* = 0.228, *P*=0.091; *r* = 0.170, *P*=0.210; *r* = 0.227, *P*=0.093).

## 4. Discussion

Glaucoma is a multifactorial process, and recent studies have speculated that choroid thickness is associated with glaucoma progression [4, 13]. The present study adds to the growing work on the relationship between choroidal thickness and PXG or that between choroidal thickness and POAG. A study has shown that macular choroidal thickness in POAG patients is not significantly different from that in healthy individuals [14]. In contrast, Cennamo et al. [15] found that the macular choroidal thickness of POAG patients measured by SD-OCT was thicker than that of healthy individuals. Egrilmez et al. [16] found that the macular choroidal thickness of PEX patients was thinner than that of POAG patients and healthy individuals. Moghimi et al. [17] argued that the choroidal thicknesses and volumes in the central subfield subfoveal region, superior quadrant, and nasal quadrant of inner rings were all significantly lower in PEX patients than in healthy individuals, while there were no significant differences in the macular choroidal thickness after adjustment for age, gender, and axial length. Our previous studies [12, 18] found that the macular choroidal thicknesses of PXG eyes, fellow eyes without PXG, and PEX eyes were thinner than those of normal eyes; that the macular choroidal thicknesses of PXG eyes (except on the temporal region) were thinner than those of fellow eyes without PXG; and that macular choroidal thickness was not significantly different between PXG eyes and PEX eyes. Our previous studies also found that the choroidal thicknesses of PEX eyes with normal IOP were thinner than those of normal eyes and that the macular choroidal thickness became progressively thinner as PXG progressed. Therefore, we speculated that the macular choroidal thickness in PEX eyes changed before the IOP increased and that risk factors for PXG might include factors unrelated to IOP that might be related to hemodynamic changes caused by the impact of exfoliative material on the vascular structure [19]. To further elucidate the effect of exfoliative material and high IOP on choroidal thickness, this study performed a comparative analysis of the choroidal thicknesses of PXG and POAG eyes. After matching all three groups of subjects for age, gender, IOP, and axial length, we found that the macular choroidal thicknesses in PXG eyes were thinner than those in POAG and normal eyes, while there was no significant difference in macular choroidal thickness between POAG eyes and normal eyes. Koz et al. [20] detected glaucomatous optic nerve damage in PEX patients with normal IOP. They speculated that the presence of the highest IOP and greatest IOP fluctuation in PEX eyes may be important factors in the progression of glaucoma and that the presence of exfoliative material may be an independent risk factor for glaucomatous optic neuropathy. Exfoliative material has been proven to accumulate in small vascular endothelial cells and pericytes and to regulate microcirculation, and the deposition of exfoliative material in blood vessels can cause insufficient circulation or occlusion, leading to ischemic changes [8]. Kose and Tekeli [21] reported that the vascular densities around the optic disc and in the macula of PXG eyes were lower than those of the POAG eyes and considered the perfusion-related injury around the optic disc and in the macula as a risk factor for the progression of glaucoma, which is faster in PXG patients. The above studies all indicated that vascular factors may play an important role in the pathogenesis of PXG.

This study still has some limitations. First, the number of patients enrolled in the study was relatively small. This study set stringent inclusion criteria, such as age and axial length, to ensure a strict match between the experimental groups and the control group. Second, due to the lack of automatic measurement software, manual delineation of the choroidal margin might have introduced some measurement errors. Third, previous studies have shown that diurnal changes in choroidal thickness occur [22]. However, OCT examinations of different participants were performed at random hours, which introduced some errors. Fourth, patients with a history of hypertension were not included in this study, and the influences of systolic blood pressure, diastolic blood pressure, and ocular perfusion pressure on choroidal thickness were not evaluated.

## 5. Conclusions

The macular choroidal thicknesses and volumes (inferior and temporal) in PXG patients were thinner and smaller than those in POAG patients and healthy individuals. The role of choroidal thickness changes in the course of PXG remains unclear. The impact of macular choroidal thickness on glaucoma needs to be further investigated in extensive multicenter trials.

## Figures and Tables

**Table 1:** Baseline characteristics of the study groups are shown in three groups.

Groups	Eyes (*n*)	Gender (*n*)	Age (years)	AL (mm)	MD (dB)	Time (days)	Pretreatment IOP (mmHg)	Posttreatment IOP (mmHg)	Antiglaucoma medications (*n*)
		M/F							
PXG	50	25/25	75.36 ± 6.75	23.19 ± 0.99	−16.76 ± 9.53	58.70 ± 50.56	30.36 ± 7.82	16.30 ± 2.97	2.78 ± 1.02
POAG	56	22/34	73.66 ± 6.23	23.26 ± 0.88	−14.66 ± 8.98	65.75 ± 49.92	27.43 ± 8.11	16.16 ± 2.37	2.69 ± 1.01
Control	54	20/34	73.70 ± 4.85	23.22 ± 0.89	−1.04 ± 0.41	—	—	15.46 ± 2.52	—
*χ* ^*2*^ */F*		2.030	1.355	0.084	68.338	0.01	0.448	1.702	197.657
*P*		0.362	0.261	0.919	＜0.001	0.473	0.062	0.186	＜0.001

PXG: pseudoexfoliative glaucoma; POAG: primary open-angle glaucoma; M: male; F: female; IOP: intraocular pressure; AL: axial length; MD: mean defect; Time: duration between the first diagnosis and study enrolment.

**Table 2:** Comparisons of macular choroidal thickness by EDI-OCT in three groups.

Regions	PXG (50 eyes)		POAG (56 eyes)		Control (54 eyes)	
	TH (*µ*m)	*V* (*µ*m^3^)	TH (*µ*m)	*V* (*µ*m^3^)	TH (*µ*m)	*V* (*µ*m^3^)
CSM	181.46 ± 56.46^a^	0.14 ± 0.05	205.45 ± 74.41^b^	0.16 ± 0.06	212.13 ± 55.34	0.17 ± 0.04
NIM	164.76 ± 54.85	0.26 ± 0.09	189.25 ± 75.97	0.30 ± 0.12	186.81 ± 54.61	0.29 ± 0.09
SIM	192.10 ± 59.54	0.30 ± 0.09	207.59 ± 68.53	0.33 ± 0.11	216.35 ± 51.66	0.34 ± 0.08
TIM	182.62 ± 55.60^a^	0.29 ± 0.09^a^	202.18 ± 68.75	0.32 ± 0.11	216.28 ± 51.73	0.34 ± 0.08
IIM	169.64 ± 58.91^a^	0.27 ± 0.09^a^	203.07 ± 81.16^b^	0.32 ± 0.13^b^	195.56 ± 55.16	0.31 ± 0.08
NOM	132.48 ± 45.11	0.70 ± 0.24	155.55 ± 71.30	0.83 ± 0.38	153.81 ± 55.48	0.82 ± 0.29
SOM	191.32 ± 54.86	1.01 ± 0.29	201.89 ± 67.57	1.07 ± 0.36	211.93 ± 45.34	1.12 ± 0.24
TOM	168.72 ± 46.02^a^	0.88 ± 0.25^a^	187.14 ± 57.78	0.99 ± 0.31^b^	201.30 ± 46.34	1.06 ± 0.24
IOM	163.10 ± 54.82	0.86 ± 0.29	189.89 ± 75.44	1.00 ± 0.41	185.52 ± 50.27	0.98 ± 0.27
MM	171.80 ± 50.46^a^	0.52 ± 0.15^a^	193.56 ± 68.26^b^	0.59 ± 0.21^b^	197.74 ± 46.82	0.60 ± 0.14

PXG: pseudoexfoliative glaucoma; POAG: primary open-angle glaucoma; comparison between the PXG group and control group by LSD *t*-test, ^a^*P* < 0.05; comparison between the PXG group and POAG group by LSD *t*-test, ^b^*P* < 0.05; CSM: central subﬁeld macula; NIM: nasal inner macula; SIM: superior inner macula; IIM: inferior inner macula; TIM: temporal inner macula; NOM: nasal outer macula; SOM: superior outer macula; IOM: inferior outer macula; TOM: temporal outer macula; MM: mean macula; TH: thickness; *V*: volume. Data are expressed as mean ± standard deviation.

## Data Availability

The research data used to support the findings of this study are available from the corresponding author upon request.
